# Maternal Herpes Zoster Infection Preceding Neonatal Langerhans Cell Histiocytosis: Coincidence or Association?

**DOI:** 10.7759/cureus.70702

**Published:** 2024-10-02

**Authors:** Rahaf Alshareef, Abdulmalik Alqahtani, Shaikah Al-Aojan

**Affiliations:** 1 Department of Dermatology, Prince Sultan Military Medical City, Riyadh, SAU; 2 General Directorate of Research and Studies, Ministry of Health, Riyadh, SAU; 3 Department of Histopathology, Prince Sultan Military Medical City, Riyadh, SAU

**Keywords:** congenital self-healing reticulohistiocytosis, langerhans cell histiocytosis, neonate, shingles, varicella zoster

## Abstract

Langerhans cell histiocytosis (LCH) is a rare proliferative disorder involving bone marrow-derived antigen-presenting cells. It can present across a wide age range, from infancy to adulthood, with neonatal cases being particularly uncommon but often associated with a favorable prognosis. The factors contributing to the development of LCH remain poorly understood, though possible influences include genetic predispositions, immune dysregulation, and prenatal environmental exposures. We report a case of a female newborn diagnosed with LCH, whose mother had a history of varicella-zoster infection during pregnancy, suggesting a potential prenatal influence.

## Introduction

Langerhans cell histiocytosis (LCH) is a heterogeneous disease that affects various age groups with neonatal disease (LCH diagnosed within 28 days after birth) having an incidence of [1-2] per million live births. It is divided into single-system disease (SS-LCH) and multisystem disease (MS-LCH) depending on several organs involved. Cutaneous manifestations are the most common presenting feature of neonatal LCH [1]. Given the rarity of the disease, the pathophysiology and etiological factors triggering neonatal LCH are not well described. Furthermore, prenatal maternal history and its impact on disease development is not well understood. We report a case of LCH associated with maternal herpes zoster infection and review the available literature on prenatal maternal disease associations and potential risk factors.

## Case presentation

**Figure 1:**
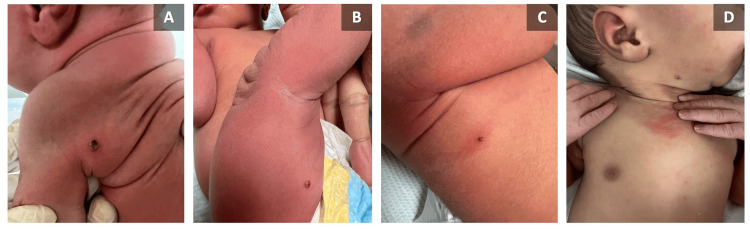
(A-D) Crusted hemorrhagic nodules over the back, thigh, and neck.

A full-term baby girl delivered via spontaneous vaginal delivery with an APGAR score of 9/9 presented with multiple hemorrhagic crusted nodules over the neck, back, and bilateral lower extremities noticed since birth (Figure 1). She had no skin detachment, erosions, bullae, necrosis, or involvement of mucous membranes. No palpable lymph nodes or hepatosplenomegaly. Darier's sign was negative. Perinatal history was unremarkable except for a maternal varicella zoster infection during the sixth month of pregnancy. The mother did not receive any treatment as lesions resolved spontaneously within one week. The mother gave a history of chickenpox infection during her childhood. 

**Figure 2:**
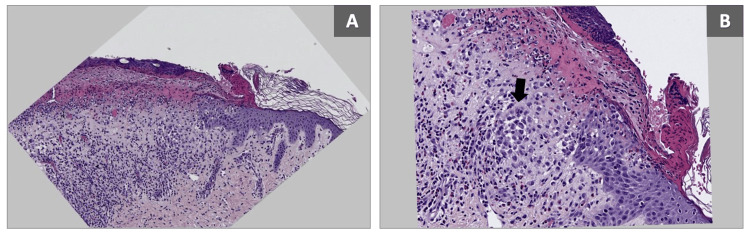
(A) Infiltration of the epidermis and superficial dermis by large cells with amphophilic cytoplasm and reniform nuclei, intermixed with eosinophils and lymphocytes (hematoxylin and eosin, original magnification 10x); (B) a black arrow points to a cluster of Langerhans cells surrounded by a mixed inflammatory infiltrate (hematoxylin and eosin, original magnification 20x).

**Figure 3:**
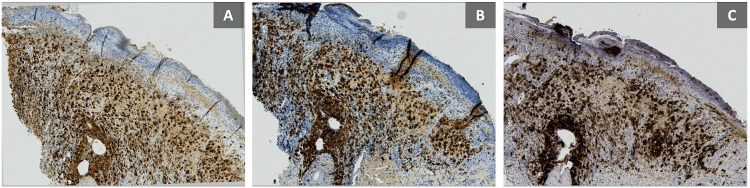
(A-C) Immunohistochemistry using S100, Langerin, and CD1a shows positive staining of large cells (original magnification 20x).

The patient underwent two skin biopsies for routine histology and tissue culture. Cultures came back to be negative for bacterial and fungal infections. Routine histology revealed an ulcerated epidermis infiltrated by a population of large cells with amphophilic cytoplasm and reniform nuclei, intermixed with eosinophils, neutrophils, and lymphocytes (Figure 2). The infiltration also involved the papillary dermis which additionally showed prominent dilated blood vessels. These large cells were found to be positive for S100, langerin, and CD1a (Figure 3). A diagnosis of LCH was made. Consultations with infectious disease and ophthalmology were done, which showed no abnormality. Skeletal survey, brain MRI, and abdominal US were also unremarkable. The lumbar puncture revealed low glucose levels, while protein and white blood cell counts were otherwise normal. Complete blood count and liver enzymes were within normal levels.

The lesions regressed completely within a few weeks with no recurrence of lesions or sequelae. No systemic associations were found during the three-month follow-up, and the patient continued to be monitored longitudinally.

## Discussion

LCH is a rare proliferative disorder of bone marrow-derived antigen-presenting cells. It is classified into two groups based on the extent of organ involvement at diagnosis: SS-LCH and MS-LCH [1,2]. Early childhood presentation is often linked to multisystem involvement, which is associated with a poorer prognosis. In contrast, SS-LCH in neonates tends to be confined to the skin (cutaneous LCH), typically presenting with a favorable prognosis due to spontaneous regression, a phenomenon known as congenital self-healing reticulohistiocytosis [1].

The clinical presentation of cutaneous LCH in neonates is variable. The most common manifestation is an erythematous vesiculopustular eruption, though other presentations such as erythematous papules, blueberry muffin rash, and oral mucosal erosions have been reported [3,4]. Given the diverse presentation, diagnosing cutaneous LCH can be challenging, as its appearance may mimic other skin conditions. For example, Johno et al. described a case of LCH presenting as a varicelliform eruption resembling the presentation of varicella infection [5], while Wee et al. reported a neonate with LCH presenting with nonspecific vesicles and papules on the first day of life. Notably, in Wee et al.'s case, the differential diagnosis initially included neonatal herpes simplex virus (HSV) infection, as the mother had a history of third-trimester genital HSV infection [6]. In our study, the patient presented with hemorrhagic nodules, and there was a history of maternal varicella zoster infection during the second trimester of pregnancy. As no prior literature has identified this as a potential risk factor for neonatal LCH, full investigations were done to rule out neonatal infections and other inflammatory disorders with similar presentation in the neonatal period.

Other prenatal and perinatal factors associated with LCH in a neonate were scantly reviewed in the literature. In a study done by Hame et al., comparing LCH patients to two control groups (children with cancer and healthy community controls). The study found that maternal urinary tract infections were more frequently reported in the LCH group compared to both community controls (relative risk [RR] = 2.64, 95% confidence interval [CI] 1.27-5.48, *P* = 0.009) and cancer controls (RR = 1.92, 95% CI, 1.09-3.36, *P* = 0.02) [7]. Bhatia et al. identified neonatal infections, solvent exposure, and a family history of thyroid disease as significant risk factors for LCH [8]. Venkatramani et al. further noted that parental occupational exposure to metals, granite, and wood dust could increase the risk of LCH [9]. Additionally, one study revealed an association between LCH development and race/ethnicity, showing that children of Hispanic mothers had a higher likelihood of developing LCH compared to non-Hispanic white children (odds ratio [OR] 1.51, 95% CI 1.02-2.25), with the risk increasing when both parents were Hispanic (OR 1.80, 95% CI 1.13-2.87) [10].

As previously stated, newborns with cutaneous SS-LCH generally have a favorable prognosis, with a 94% survival rate at five years. Thus, conservative management is often appropriate since in many cases the skin lesions typically resolve spontaneously around four months after they first appear [1]. However, patients should be closely monitored for possible complications that could occur months to years after the diagnosis with cutaneous SS-LCH such as diabetes insipidus, panhypopituitarism, and bony relapse. Therefore, regular follow-up is essential for patients with SS-LCH [1,11].

## Conclusions

Our case highlights the importance of obtaining a full maternal prenatal history, including the history of infections in patients with neonatal LCH. Identifying potential associations and their contribution to a better understanding of disease pathogenesis will result in early diagnosis and management of cases. 
